# Experimental Preeclampsia Causes Long-Lasting Hippocampal Vascular Dysfunction and Memory Impairment

**DOI:** 10.3389/fphys.2022.889918

**Published:** 2022-05-09

**Authors:** Abbie C. Johnson, Sarah M. Tremble, Marilyn J. Cipolla

**Affiliations:** ^1^ Department of Neurological Sciences, University of Vermont Larner College of Medicine, Burlington, VT, United States; ^2^ Department of Obstetrics, Gynecology, and Reproductive Sciences, University of Vermont Larner College of Medicine, Burlington, VT, United States; ^3^ Department of Pharmacology, University of Vermont Larner College of Medicine, Burlington, VT, United States; ^4^ Department of Electrical and Biomedical Engineering, University of Vermont College of Engineering and Mathematical Sciences, Burlington, VT, United States

**Keywords:** preeclampsia, hippocampal arterioles, memory impairment, dementia, hippocampus

## Abstract

Preeclampsia (PE) is a hypertensive disorder of pregnancy that is associated with memory impairment, cognitive decline and brain atrophy later in life in women at ages as young as early-to-mid 40 s. PE increases the risk of vascular dementia three-fold, however, long-lasting effects of PE on the vasculature of vulnerable brain regions involved in memory and cognition, such as the hippocampus, remain unknown. Here, we used a rat model of experimental PE (ePE) induced by maintaining rats on a 2% cholesterol diet beginning on day 7 of gestation to investigate hippocampal function later in life. Hippocampal-dependent memory and hippocampal arteriole (HA) function were determined in Sprague Dawley rats 5 months after either a healthy pregnancy or ePE (*n* = 8/group). Rats that had ePE were hypertensive and had impaired vasoreactivity of HAs to mediators involved in matching neuronal activity with local blood flow (i.e., neurovascular coupling). ePE rats also had impaired long-term memory, but not spatial memory. Thus, this model of ePE mimics some of the long-lasting cardiovascular and cognitive consequences that occur in women who previously had PE. These findings suggest endothelial and vascular smooth muscle dysfunction of HAs were present months after PE that could impair hippocampal neurovascular coupling. This represents a novel vascular mechanism by which PE causes early-onset dementia.

## Introduction

Preeclampsia (PE), a hypertensive disorder of pregnancy, complicates up to 10% of pregnancies world-wide and is a leading cause of maternal and fetal morbidity and mortality (MacKay et al., 2001; Steegers et al., 2010; Miller, 2019). PE affects several maternal organ systems, including the cerebral circulation and the brain. Although PE is a disease of pregnancy, its effects are long-lasting. In fact, PE is a major risk factor for cardiovascular and cerebrovascular disease later in life (e.g., hypertension, stroke) and increases the risk for dementia, specifically vascular dementia (Basit et al., 2018; Miller, 2019; Miller and Leffert, 2019). Importantly, women who had pregnancies complicated by PE report cognitive deficits, including problems with memory, and have brain atrophy at relatively young ages (e.g., in their 40 s) (Brussé et al., 2008; Fields et al., 2017; Siepmann et al., 2017; Dayan et al., 2018; Elharram et al., 2018). Further, the changes in gray matter volume and white matter lesion burden occur as a function of time from pregnancy, suggesting that former PE continues to progressively damage the brain long after the affected pregnancy (Siepmann et al., 2017). Understanding long-lasting effects of previous PE pregnancies on the vasculature of brain regions critically involved in memory and cognition is essential in order to develop therapeutic strategies to ameliorate memory dysfunction occurring after PE and improve quality of life in these relatively young women.

Mechanisms by which PE exerts long-lasting and potentially progressive deleterious effects on cognitive function are unclear, but likely involve the hippocampus given its central role in memory and cognition. The hippocampus is a bilateral structure located deep in the temporal lobes of humans. Importantly, it is more susceptible to hypoxic/ischemic injury than cortical brain regions (Nikonenko et al., 2009), making the small hippocampal arterioles (HAs) responsible for perfusing the hippocampus central to maintaining neurocognitive health. These arterioles are involved in the rapid cascade of events that match neuronal metabolic demand with appropriate local blood flow. This dynamic communication between neurons, glia, and the vasculature, termed neurovascular coupling, is critical to neuronal health, and has been shown to be impaired in the cerebral cortex in women that had PE (Martens et al., 2009; Janzarik et al., 2014). Importantly, we have previously shown that HAs were smaller and stiffer, with a limited vasodilatory capacity and impaired vasodilation to mediators of neurovascular coupling in an experimental rat model of PE (Johnson and Cipolla, 2017). While these changes occurred during the affected pregnancy, it is not clear whether this hippocampal vascular dysfunction persists into the post-partum period that could contribute to long-lasting memory dysfunction after PE.

In the current study, we used an established rat model of experimental PE (ePE) to investigate the impact of PE on hippocampal function 5 months post-partum. We assessed hippocampal-dependent memory and studied the function of isolated and pressurized HAs to test the hypothesis that ePE causes long-lasting hippocampal vascular dysfunction that is associated with impaired memory function. To investigate potential mechanisms by which ePE disrupts hippocampal function, vascular responses to mediators of neurovascular coupling were determined, including activation of small- and intermediate-conductance calcium-activated potassium (SK_Ca_/IK_Ca_) channels, inward-rectifying potassium (K_IR_) channels, and nitric oxide (NO).

## Materials and Methods

### Animals

Experiments were conducted using 16 female Sprague Dawley rats purchased from Charles River, Canada. Rats (14–16 weeks old) were purchased on gestational day (gd) 7 of pregnancy and were single housed with environmental enrichment in the University of Vermont Animal Care Facility, an Association for Assessment and Accreditation of Laboratory Animal Care International (AAALAC) accredited facility. Rats were maintained on a 12-h light/dark cycle and allowed access to food and water ad libitum. All procedures were approved by the Institutional Animal Care and Use Committee and conducted in accordance with the National Institutes of Health (NIH) Guide for the Care and Use of Laboratory Animals. All rats were single housed with enrichment for the duration of pregnancy. Pups were weaned on post-natal day 21, and post-partum rats were housed in pairs with enrichment 1 week later and remained housed in pairs until experimental use 5 months post-partum. All euthanasia was under isoflurane anesthesia according to NIH guidelines.

### Model of Experimental Preeclampsia

In order to investigate long-lasting cognitive and cerebrovascular consequences of PE, we used a model of ePE that involves maintaining pregnant rats (*n* = 8) on a high cholesterol diet beginning on gd 7 (Prolab 3,000 rat chow with 2% cholesterol and 0.5% sodium cholate; Scotts Distributing Inc., Hudson, NH, United States) (Schreurs and Cipolla, 2013; Schreurs et al., 2013; Johnson and Cipolla, 2017; Johnson and Cipolla, 2018). This model has been previously characterized during pregnancy and shown to induce several PE-like symptoms, including maternal endothelial dysfunction, elevated blood pressure, dyslipidemia, fetal growth restriction and placental disease (Schreurs and Cipolla, 2013; Schreurs et al., 2013; Johnson and Cipolla, 2017; Johnson and Cipolla, 2018). Post-Partum ePE rats were maintained on the high cholesterol diet until experimental use. As 1 month of age in an adult rat is equivalent to ∼ 3 years in adult humans, 5 months after ePE is approximately 15 years after PE (Sengupta, 2013). Thus, studying hippocampal function 5 months post-partum mimics the time frame of early-onset cognitive impairment and brain atrophy that occur in women with a history of PE (Fields et al., 2017; Siepmann et al., 2017; Dayan et al., 2018; Elharram et al., 2018).

### Behavioral Tests of Memory Function

To determine potentially long-lasting effects of ePE on hippocampal-dependent cognitive function, long-term memory and spatial memory were tested using a novel object recognition (NOR) task and continuous Y maze task, as we have done previously (Johnson et al., 2020; Johnson et al., 2021). Briefly, 2 weeks prior to isolated vessel experiments ( ∼ 4.5 months post-partum), rats that had a healthy pregnancy (Post-Partum Preg, *n* = 8) and rats that had ePE (Post-Partum ePE, *n* = 8) acclimated to the behavioral room for 1 h prior to testing. For the NOR task, each rat was allowed to habituate to the open field arena for 5 min, followed by acquisition period exploring two identical objects for 10 min (Ballarini et al., 2009). Twenty-four h later, rats were placed in the same arena containing one familiar and one novel object. The time spent investigating both the novel and familiar objects was used to calculate a recognition index as a measure of long-term memory function. For the continuous Y maze task, rats were allowed to freely explore a Y maze for 8 min. Y maze tasks were video recorded to allow for quantification of spontaneous alternation behavior and total arm entries that were used to calculate an alternation index as a measure of spatial memory (Hughes, 2004; Johnson et al., 2020). All behavioral analyses were completed by a reviewer that was blinded to group.

### Non-Invasive Blood Pressure Measurements

To determine if ePE caused hypertension in the post-partum period, blood pressures were measured non-invasively *via* the tail-cuff method in Post-Partum Preg and Post-Partum ePE rats, as described previously (Schreurs and Cipolla, 2013; Schreurs et al., 2013; Li et al., 2020). Briefly, after behavioral tasks, rats were allowed to acclimate to the environment in their home cages for 1 h prior to measurement. Rats were placed in individual acrylic tube holders on a heated platform and blood pressure cuffs placed at the base of the tail (CODA-8 System, Kent Scientific, Torrington, CT, United States). Systolic and diastolic blood pressures were measured and mean arterial pressure (MAP) estimated by 1/3 systolic +2/3 diastolic pressure. To avoid stress-induced elevations in blood pressure associated with being handled and restrained in the holder, rats were trained in the tail-cuff apparatus for 3 days prior to measurements. Further, to avoid any interference of blood pressure measurements on memory tasks, tail-cuff measurements were taken after all behavioral tests were completed.

### Isolated Hippocampal Arteriole Function

To understand the potential role of the hippocampal vasculature in hippocampal dysfunction after ePE, HAs supplying the dorsal hippocampus were isolated and studied in a pressurized arteriograph system, as previously described (Johnson and Cipolla, 2016; Johnson et al., 2020). After behavioral testing and blood pressure measurements were completed, rats that were 5 months post-partum were decapitated under deep isoflurane anesthesia (3% in oxygen). Blood glucose was measured at the time of decapitation using a glucometer and brains were immediately removed and placed in cold, oxygenated artificial cerebrospinal fluid (aCSF). HAs were carefully dissected, mounted on glass cannulas and pressurized in an arteriograph chamber (Living Systems Instrumentation, Burlington, VT, United States) (Johnson and Cipolla, 2016; Johnson et al., 2020). HAs were equilibrated at 20 mmHg for 1 h, after which intravascular pressure was increased to 120 mmHg in 20 mmHg increments to measure myogenic tone and reactivity. Lumen diameter and wall thickness were recorded at each intravascular pressure once stable. Pressure was then returned to 60 mmHg for the remainder of the experiment. To investigate endothelial and vascular smooth muscle function of HAs to mediators of neurovascular coupling, reactivity to various pharmacological agents was measured: NS309 (10^–8^—10^–5^ M), an SK_Ca_/IK_Ca_ channel agonist; extracellular K^+^ (3–40 mM) to determine inward rectifying potassium (K_IR_) channel function; and sodium nitroprusside (SNP, 10^–8^—10^–5^ M), a NO donor. At the end of each experiment, aCSF was replaced with aCSF containing zero calcium, papaverine (10^–4^ M) and diltiazem (10^–5^ M) to fully relax the vascular smooth muscle, and passive structural measurements made within the pressure range of 5–120 mmHg.

### Drugs and Solutions

Diltiazem was purchased from MP Biomedicals (Santa Ana, CA, United States), and all other compounds, including NS309, KCl, SNP, papaverine, and those used to make aCSF were purchased from Sigma Aldrich (St. Louis, MO, United States). Stock solutions of SNP, papaverine and diltiazem were made weekly and stored at 4°C until use. NS309 stock solution was aliquoted and stored at -20°C until use. aCSF contained (mM): NaCl 122.0, NaHCO_3_ 26.0, NaH_3_PO_4_ 1.25, KCl 3.0, MgCl_2_ 1.0, and CaCl_2_ 2.0. aCSF with higher concentrations of KCl (5, 10, 15, 20, 30, 40 mM) were made with reduced amounts of NaCl to maintain constant osmolality. Buffer solutions were made each week and stored without glucose at 4°C. Glucose was added (4.0 mM) immediately prior to each experiment. Zero calcium aCSF was made similarly, omitting the CaCl_2_ and with the addition of 0.5 mM EGTA. aCSF was aerated with 5% CO_2_, 10% O_2_ and 85% N_2_ to maintain pH at 7.40 ± 0.05 and the temperature within the arteriograph chamber was maintained at 37.0 ± 0.1°C throughout the experiments.

### Data Calculations

To determine memory function, recognition index was calculated using the following formula: Time_Novel_/Time_Total_ where Time_Novel_ is the amount of time (sec) a rat spent investigating the novel object and Time_Total_ is the total time (sec) that rat spent investigating both objects. Spontaneous alternation index was calculated using the following formula: [# Alternations/(# Arm Entries—2)]. For isolated HA experiments, percent myogenic tone was calculated using the following equation: % Tone = [1—(φ_active_/φ_passive_)] x 100%; where φ_active_ is the diameter under active conditions and φ_passive_ is the diameter under fully relaxed conditions at a specific intravascular pressure. Percent reactivity to NS309 and SNP was calculated from the equation: % Reactivity = [(φ_dose_–φ_baseline_)/(φ_passive_–φ_baseline_)] x 100%; where φ_dose_ is the diameter of the vessel after treatment with a specific concentration of drug and φ_baseline_ is the starting diameter before any drug treatment. Percent change of lumen diameter in response to different concentrations of K^+^ were calculated from baseline diameter using the equation: % Change = [(φ_dose_–φ_baseline_)/(φ_baseline_)] x 100%. Outer diameter (φ_outer_) was calculated at each pressure by the equation: φ_outer_ = φ_inner_ + 2 WT; where φ_inner_ is the inner diameter of the vessel fully relaxed and WT is the measured wall thickness. Cross-sectional area (CSA) was calculated by the equation: CSA = π(φ_outer_/2)^2^—π(φ_inner_/2)^2^ at each intravascular pressure. Percent distensibility was calculated using the equation: % Distensibility = [(φ_passive_–φ_5_)/φ_5_] x 100%; where φ_5_ is the inner diameter of the fully relaxed vessel at an intravascular pressure of 5 mmHg.

### Statistical Analyses

The number of animals used in each experiment was determined by a statistical power calculation using a two-sided 95% confidence interval for a single mean and 1—β of 0.80 based on previous studies using similar methodology (Johnson and Cipolla, 2017; Johnson et al., 2020). Results are presented as mean ± SEM and a *p*-value of <0.05 was considered significant. Using GraphPad Prism 8.0 software (GraphPad Software Inc., La Jolla, CA), differences between groups were determined using a two-sided Mann-Whitney test. Rats were randomized to treatment group, and the order of experiments were randomized using an online randomization tool (Random.org). All experiments and data analyses were completed blinded to group.

## Results

### Physiological Parameters of Post-Partum Preg and Post-Partum ePE Rats

All rats had similar litter sizes regardless of ePE status, with both healthy pregnant rats and rats with ePE birthing 13 ± 1 pups. Five months post-partum, rats that had ePE weighed significantly more than rats that had a normal pregnancy; however, blood glucose levels were similar (Table 1). Post-Partum ePE rats were hypertensive, with elevated systolic, diastolic and MAP compared to Post-Partum Preg rats (Table 1), indicating ePE had long-lasting cardiovascular effects that were similar to what occurs in formerly PE women.

**TABLE 1:** Physiological parameters of female rats 5 months post-partum that had either a healthy pregnancy (Post-Partum Preg) or that had experimental preeclampsia (Post-Partum ePE).

	Body weight (g)	Blood glucose (mg/dl)	Systolic blood pressure (mmHg)	Diastolic blood pressure (mmHg)	Mean arterial pressure (mmHg)
Post-Partum Preg (*n* = 8)	373 ± 9	151 ± 14	129 ± 7	97 ± 4	106 ± 4
Post-Partum ePE (*n* = 8)	407 ± 10^a^	134 ± 7	142 ± 2^a^	106 ± 3	117 ± 3^a^

a *p* < 0.05 by Mann-Whitney test.

### ePE Disrupted Memory Function 5 Months After Pregnancy

**FIGURE 1:**
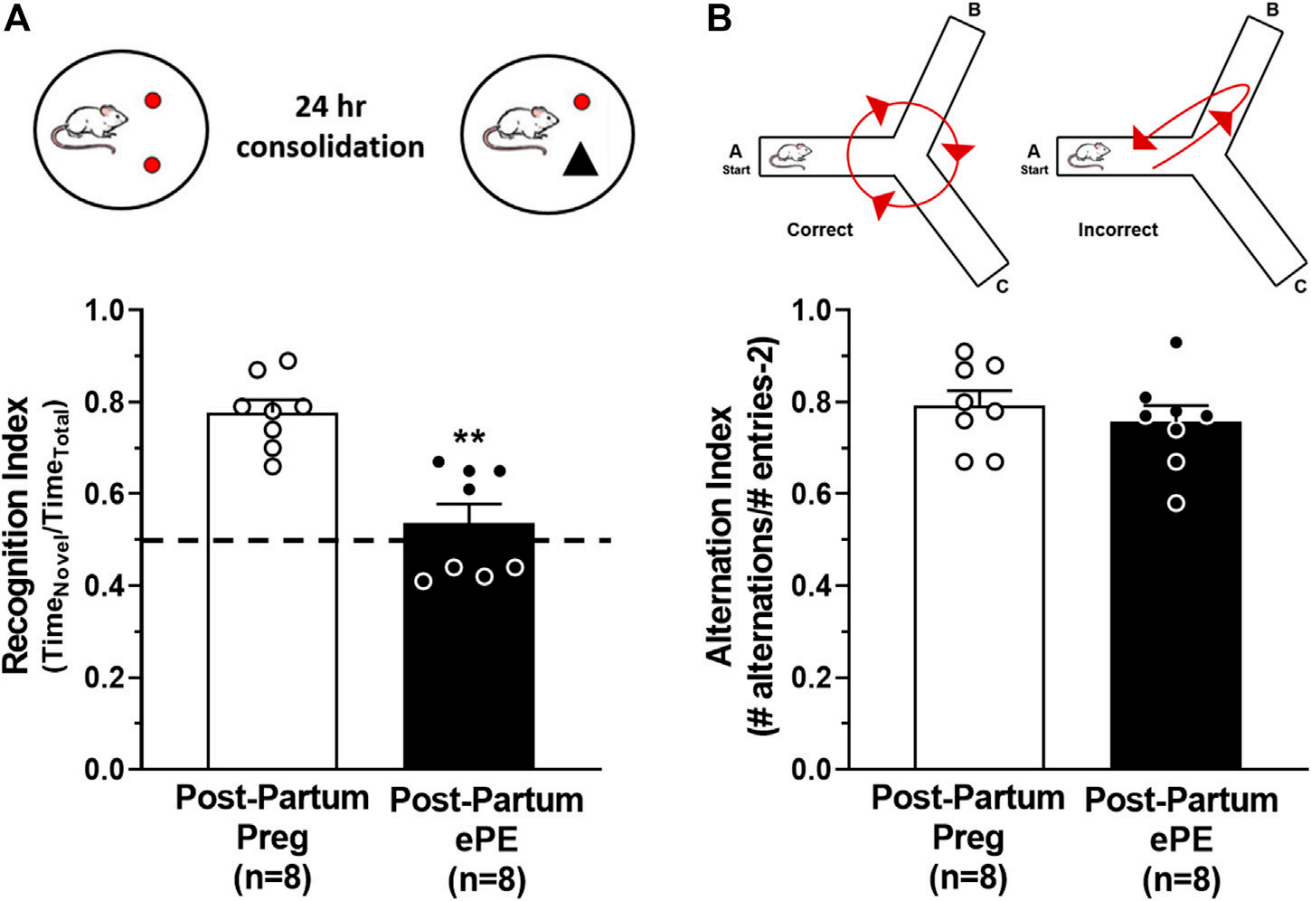
Behavioral assessments of memory function 5 months after either a healthy pregnancy (Post-Partum Preg) or experimental preeclampsia (Post-Partum ePE). **(A)** Illustration of the novel object recognition (NOR) task paradigm consisting of a 24-h consolidation period to allow for assessment of long-term memory function. Recognition Index was decreased in Post-Partum ePE rats compared to Post-Partum Preg rats. **(B)** Illustration of the inherent spontaneous alternation behavior of cognitively intact rodents preferring to alternate between Y maze arms (left) and arm reversals occurring in error (right) as an assessment of spatial memory function. Alternation Index was similar between Post-Partum Preg and Post-Partum ePE rats. Data are mean ± SEM, **p < 0.01 by Mann-Whitney test.

Formerly PE women report cognitive dysfunction at relatively young ages, including problems with memory (Brussé et al., 2008; Elharram et al., 2018). To investigate the long-term effect of ePE on hippocampal function, we used behavioral tasks to assess memory in rats 5 months after either a healthy pregnancy or ePE. An object recognition task was used to quantify the amount of time spent investigating a novel and a familiar object and a recognition index was calculated as a measure of long-term memory function. A rodent that is cognitively intact will spend the majority of the time investigating the novel object and have a recognition index >0.50. However, a rat with impaired memory will not remember the familiar object and spend closer to equal amounts of time investigating both objects (recognition index ∼ 0.50) (Broadbent et al., 2010; Quillfeldt et al., 2016). Figure 1A shows that Post-Partum ePE rats had a significantly reduced recognition index compared to Post-Partum Preg rats, indicating impaired long-term memory function 5 months after ePE. As the hippocampus has a predominant role in spatial navigation, we also used a continuous Y maze task to calculate alternation index and assess spatial memory function. Rats with intact spatial memory will exhibit spontaneous alternation behavior: they remember the arm of the maze they were recently in and naturally alternate between maze arms. A rat with impaired spatial memory will reverse directions and return to the maze arm they just visited (Figure 1B) (Lalonde, 2002; Hughes, 2004). Interestingly, both groups performed similarly well in the continuous Y maze task, with alternation indices at ∼ 0.8 for each group (Figure 1B). Together, these findings suggest that ePE differentially affects long-term memory without disrupting spatial memory well into the post-partum period.

### Long-Lasting HA Dysfunction in Rats That had ePE

**FIGURE 2:**
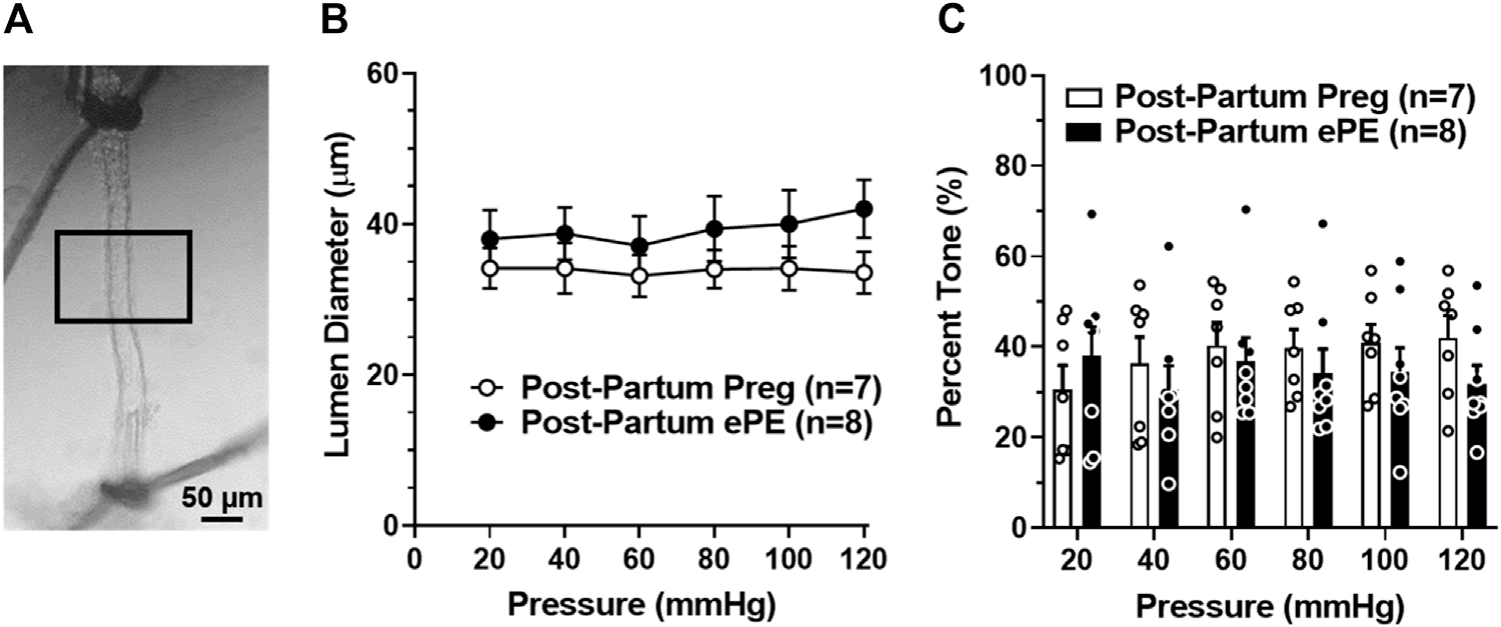
Active lumen diameters and myogenic tone of hippocampal arterioles (HAs). **(A)** Photomicrograph of a pressurized HA in an arteriograph chamber with 40% myogenic tone. Boxed inset denotes the section of arteriole where lumen diameters and wall thickness measurements were made. Scale bar is 50 µm. **(B)** Lumen diameters of HAs from Post-Partum Preg and Post-Partum ePE rats were similar across the intravascular pressure range of 20–120 mmHg. **(C)** Calculated percent myogenic tone was similar between groups at all pressures investigated. Data are mean ± SEM.

To determine if HA function was disrupted 5 months after ePE potentially contributing to impaired memory function, freshly isolated and pressurized arterioles that supply the dorsal hippocampus were studied (Figure 2A) (Johnson and Cipolla, 2017; Johnson et al., 2020). One Post-Partum Preg rat was excluded due to technical difficulties. Lumen diameters remained relatively unchanged across the intravascular pressure range studied (Figure 2B) and HAs from both groups developed substantial pressure-induced myogenic tone at 20 mmHg that was maintained similarly across the intravascular pressure range studied (Figure 2C). Lumen diameter increased and myogenic tone decreased at 120 mmHg in arterioles from Post-Partum ePE rats, although this was not statistically different from Post-Partum Preg rats.

**FIGURE 3:**
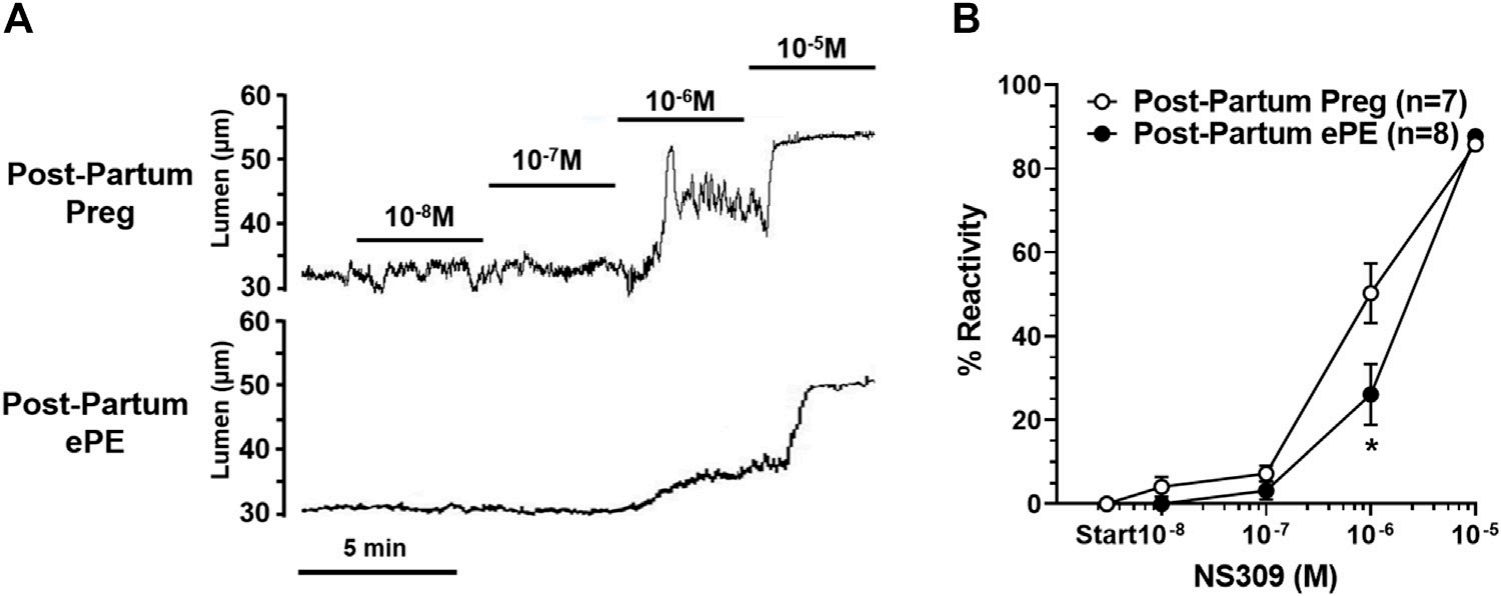
Reactivity of hippocampal arterioles (HAs) to small- and intermediate-conductance calcium-activated (SK_Ca_/IK_Ca_) channel activation. **(A)** Representative lumen diameter tracings of HAs from one Post-Partum Preg and one Post-Partum ePE rat during the concentration response curve to the SK_Ca_/IK_Ca_ channel agonist NS309. Solid lines represent cumulative doses of NS309 from 10^−8^ M to 10^−5^ M. **(B)** Percent reactivity of HAs in response to increased concentrations of NS309 was shifted to the right in arterioles from Post-Partum ePE rats compared to those from Post-Partum Preg rats. Data are mean ± SEM, *p < 0.05 by Mann-Whitney test.

**FIGURE 4:**
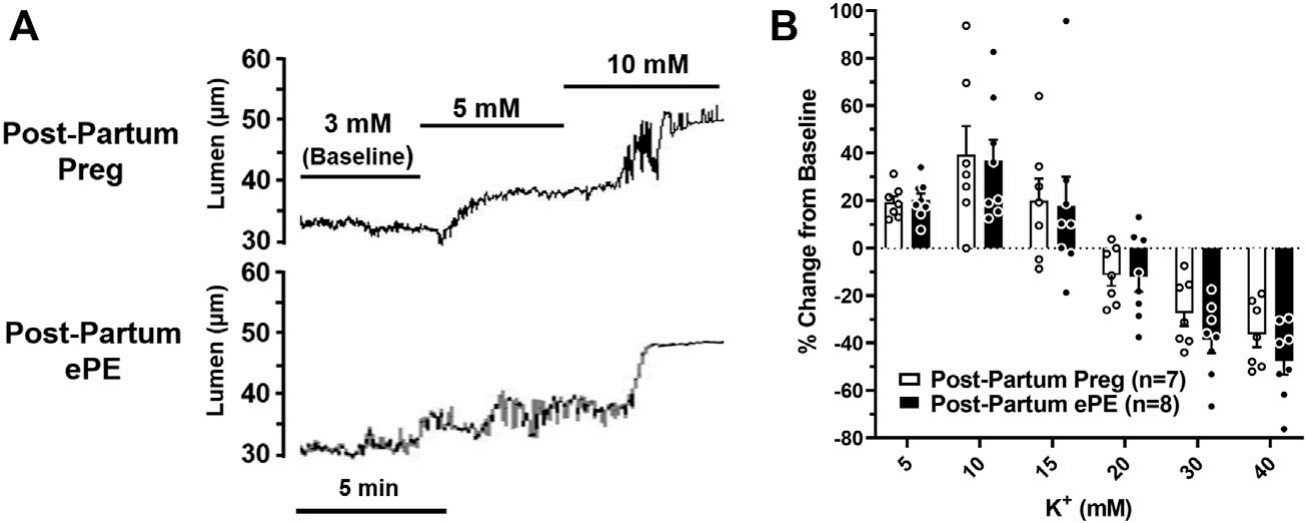
Reactivity of hippocampal arterioles (HAs) to increased extracellular K^+^ concentrations. **(A)** Representative lumen diameter tracing of HAs from a Post-Partum Preg rat and a Post-Partum ePE rat during exposure to increased extracellular K^+^ concentrations from 3–10 mM. **(B)** Percent change in diameter from baseline (3 mM K^+^) was similar between groups at all concentrations of extracellular K^+^ investigated. Data are mean ± SEM.

We investigated vasoreactivity of HAs to mediators involved in neurovascular coupling, including SK_Ca_/IK_Ca_ channels, K_IR_ channels, and NO. SK_Ca_/IK_Ca_ channels are expressed in the endothelium of intraparenchymal arterioles, including HAs that upon activation cause endothelial-dependent vasodilation (Ledoux et al., 2006). Figure 3A shows representative lumen diameter traces of HAs in response to SK_Ca_/IK_Ca_ channel activation with NS309 as an assessment of endothelial function. Lumen diameters increased in a concentration-dependent manner in both groups and percent reactivity was significantly reduced in HAs from Post-Partum ePE rats at 10^−6^ M NS309, although was similar between groups at the highest concentration (Figure 3B), suggesting endothelial dysfunction in the hippocampus of rats that had ePE. Elevated extracellular K^+^ up to ∼ 15 mM activates K_IR_ channels resulting in vasodilation of cerebral intraparenchymal arterioles, including HAs (Knot et al., 1996; Filosa et al., 2006; Longden and Nelson, 2015; Johnson and Cipolla, 2017). However, higher concentrations of extracellular K^+^ depolarize vascular smooth muscle cells and activate voltage-operated calcium channels causing vasoconstriction (Sobey, 2001). Figure 4A shows representative original lumen diameter tracings of HAs from one Post-Partum Preg and Post-Partum ePE rat during elevations in extracellular K^+^ from 3–10 mM. HAs from both groups dilated similarly within the concentration range 5–15 mM extracellular K^+^ (Figure 4B). When extracellular K^+^ was increased >15 mM, HAs decreased in diameter and constricted in a concentration-dependent manner. HAs from Post-Partum ePE rats constricted to a greater extent than arterioles from Post-Partum Preg rats at 30 and 40 mM K^+^ but this did not reach statistical significance (Figure 4B).

**FIGURE 5:**
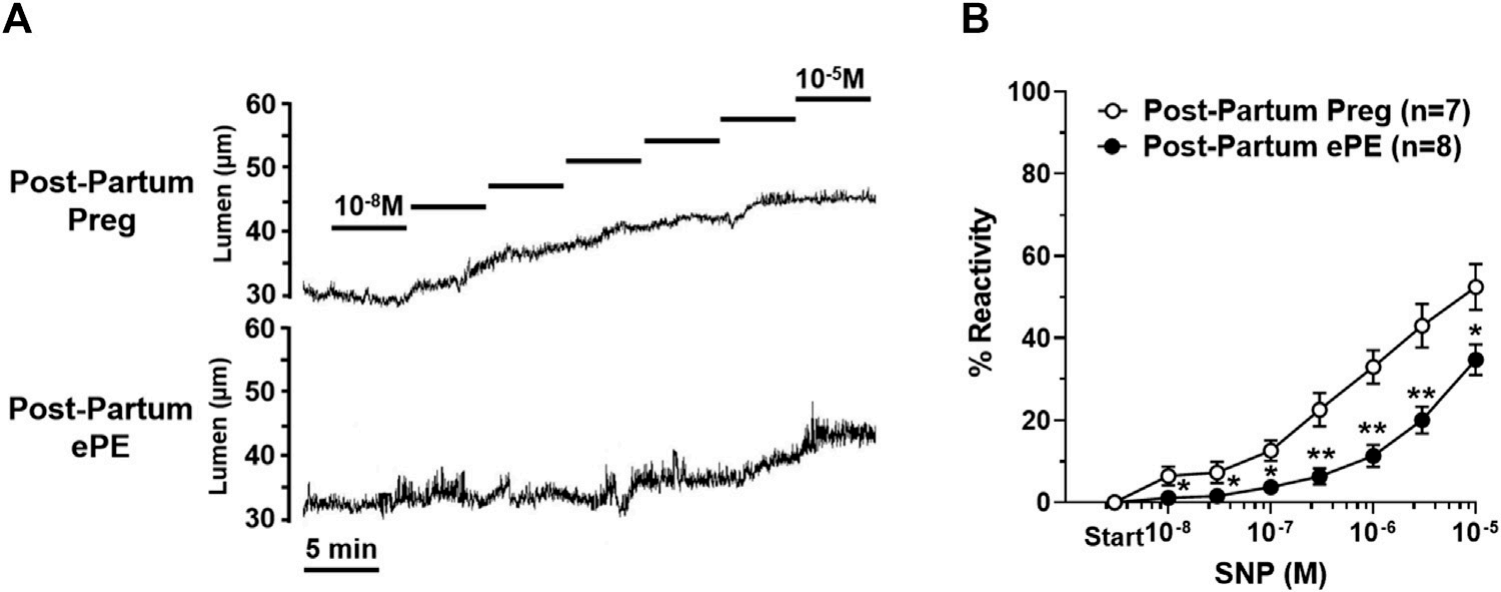
Reactivity of hippocampal arteriole (HA) vascular smooth muscle to nitric oxide (NO). **(A)** Representative lumen diameter tracings of HAs from a Post-Partum Preg rat and a Post-Partum ePE rat during the concentration response curve to NO donor SNP. **(B)** HAs from rats that had ePE were less vasoreactive to increasing concentrations of SNP compared to arterioles from Post-Partum Preg rats. Data are mean ± SEM, **p < 0.01; *p < 0.05 by Mann-Whitney test.

Sodium nitroprusside (SNP) is an endothelial-independent NO donor that relaxes vascular smooth muscle to cause vasodilation and is considered the primary mediator of neurovascular coupling in the hippocampus (Lourenço et al., 2014; Lourenço et al., 2018). HAs from both groups vasodilated in response to increasing concentrations of SNP, as shown in representative lumen diameter traces in Figure 5A. However, SNP had less of a vasodilatory effect in HAs from Post-Partum ePE rats, with vasoreactivity being substantially blunted in response to SNP compared to arterioles from rats that had a healthy pregnancy (Figure 5B).

**FIGURE 6:**
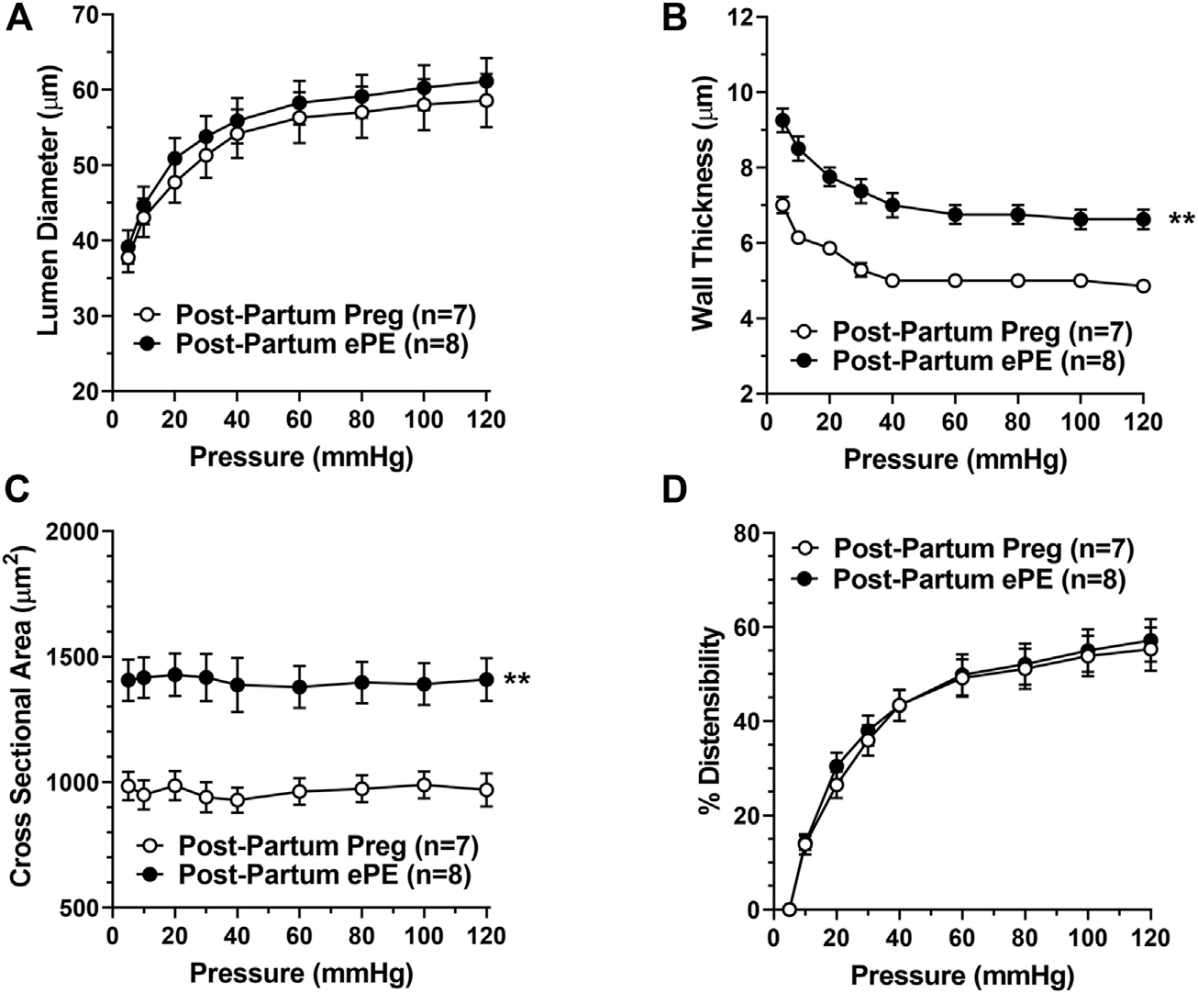
Passive structural and biomechanical properties of hippocampal arterioles (HAs) 5 months after either a healthy pregnancy or experimental preeclampsia (ePE). **(A)** Lumen diameters of fully relaxed HAs across the intravascular pressure range 5–120 mmHg were of similar size between groups. **(B)** Wall thickness and **(C)** cross-sectional area were increased in HAs from Post-Partum ePE rats compared to Post-Partum Preg rats at all pressures studied. **(D)** Distensibility of HAs was similar between groups. Data are mean ± SEM, **p < 0.01 by Mann-Whitney test.

Figure 6 shows the structural and biomechanical properties of HAs from both groups. HAs from Post-Partum ePE and Post-Partum Preg rats were similar in lumen size under fully relaxed conditions (Figure 6A). However, HAs from Post-Partum ePE rats underwent hypertrophic remodeling, and had increased vascular wall thickness and cross-sectional area (CSA) compared to rats that had a healthy pregnancy (Figures 6B,C). Lastly, distensibility of HAs was similar between groups (Figure 6D).

## Discussion

Women with a history of PE are at increased risk for cardiovascular and cerebrovascular disease later in life and often exhibit cognitive decline as early as in their 40 s (Brussé et al., 2008; Basit et al., 2018; Dayan et al., 2018; Miller, 2019). Further, women that had PE have a three-fold increase in the risk for vascular dementia, indicating potential long-lasting effects on the cerebrovasculature (Basit et al., 2018). In the current study, we found that rats that had ePE were hypertensive well into the post-partum period and had impaired endothelial and vascular smooth muscle function in HAs that was associated with impaired hippocampal-dependent long-term memory function compared to rats that had a healthy pregnancy. This is the first study that we are aware of to investigate hippocampal vascular dysfunction as a novel underlying mechanism of memory decline occurring after PE. These findings broaden our understanding of long-lasting consequences of PE and suggest the hippocampal vasculature may hold therapeutic potential in protecting the hippocampus after PE to prevent memory dysfunction in these relatively young women.

The control of local blood flow occurs at the level of pre-capillary arterioles and upstream penetrating arterioles, making HAs critical in maintaining basal perfusion of the hippocampus and protecting neurocognitive health (Fernández-Klett et al., 2010). Through their involvement in neurovascular coupling, pre-capillary arterioles are also centrally involved in neuronal activity-dependent changes in local blood flow (Fernández-Klett et al., 2010; Iadecola and Gottesman, 2019). In the hippocampus, neuronally-derived NO diffuses to surrounding microvasculature to relax vascular smooth muscle and is considered the primary mechanism by which local vasodilation occurs during hippocampal neuronal activity (Lovick et al., 1999; Lourenço et al., 2014). In the current study, the vasodilatory response of HAs to the NO donor SNP was blunted in arterioles from rats that had ePE, suggesting a limited ability of arterioles to respond to neuronal activity to increase local blood flow. Further, HAs from ePE rats had modestly impaired endothelial function, with impaired vasodilation in response to SK_Ca_/IK_Ca_ channel activation. These potassium channels are involved in conducting local vasodilation to upstream arterial segments to reduce vascular resistance and increase local blood flow, a critical mechanism to match neuronal metabolic demand with appropriate blood flow (Hannah et al., 2011; Longden and Nelson, 2015; Povlsen et al., 2016; Guerra et al., 2018). These findings suggest that two critical aspects of neurovascular coupling may be impaired in the hippocampus after ePE: the ability for neuronal activity to generate a local vasodilation, and the ability of the vasculature to conduct that vasodilation upstream to increase local blood flow. In cortical brain regions, arteriole dysfunction impairs neurovascular coupling and contributes to cognitive decline in models of Alzheimer’s disease (Girouard and Iadecola, 2006; Povlsen et al., 2016). In fact, we have previously shown that cortical penetrating arterioles from ePE rats have impaired conducted vasodilation late in gestation, suggesting neurovascular coupling may be impaired in the cortex during the affected pregnancy (Johnson and Cipolla, 2018). However, we did not compare cortical penetrating arteriole function to hippocampal arteriole function in the current study, and therefore we do not know if the long-lasting effects of ePE are region-specific and only affects HAs, or have a global effect on the cerebrovasculature in multiple/all brain regions. Regardless, we speculate that endothelial and vascular smooth muscle dysfunction after ePE results in neurovascular un-coupling in the hippocampus that impairs neuronal function and contributes to early-onset memory dysfunction.

Our previous study reported that ePE caused HAs to undergo structural remodeling, becoming both smaller and stiffer during pregnancy (Johnson and Cipolla, 2017). Interestingly, in the current study, there were no differences in lumen size between HAs from rats that had ePE and those that had a healthy pregnancy 5 months post-partum, suggesting the ePE-induced inward remodeling was transient and reverses throughout the post-partum period. Further, there were no differences in myogenic tone, which together with being structurally similar in size suggests basal hippocampal perfusion is likely unaffected 5 months after ePE. Therefore, it seems that long-lasting hemodynamic consequences of ePE most likely impair neuronal activity-dependent changes in local hippocampal blood flow rather than basal blood flow. Whether structural remodeling of HAs occurs as a function of time/age such as that which can occur in response to chronic hypertension that may affect hippocampal perfusion later in life remains unclear.

There is a substantial body of literature investigating the impact of PE on cognitive function of the offspring of rodent models of PE, however, fewer studies have investigated maternal cognitive function after PE. Given that women who had PE experience cognitive issues, including problems with memory, within a few years after pregnancy, understanding maternal cognitive consequences is important. Here, we show that rats that had ePE had impaired long-term memory function 5 months post-partum. We previously showed ePE rats and healthy pregnant rats performed similarly well in the NOR task assessing long-term memory late in gestation, indicating that memory decline associated with ePE does not begin until the post-partum period (Johnson et al., 2021). Interestingly, spatial memory was unaffected by ePE in the current study. It is not clear from the current study why long-term memory was differentially affected after ePE since both tasks largely involve the hippocampus but could be due to regional involvement and susceptibilities of certain tasks to disruption. For example, a study investigating the contribution of the hippocampus to spatial memory and object memory reported larger dorsal hippocampal lesions were necessary to impair object recognition than spatial recognition (Broadbent et al., 2004). These findings suggest spatial memory is more susceptible to disruption than object recognition that is in contrast to what we found in the current study. However, such studies use robust hippocampal lesions and neuronal loss to determine regional-dependent task involvement (Duva et al., 1997; Broadbent et al., 2004; Broadbent et al., 2010), whereas little is known about the relationship between hippocampal vascular dysfunction and different memory tasks. Regardless, here we show that dorsal hippocampal vascular dysfunction after ePE is associated with impaired long-term memory (object recognition) but not spatial memory. If HA dysfunction persists or worsens with time, it is possible that spatial memory and other aspects of cognitive function may also become impaired later in life.

A recent study investigating maternal cognitive function after PE in a mouse model reported findings that contradict those of the current study. Trigiani et al. (2021) investigated post-partum cognitive function and cerebral hemodynamics in a transgenic mouse model overexpressing human angiotensinogen and renin (R^+^A^+^ model of PE) (Trigiani et al., 2021). They showed that hippocampal-dependent memory function remained unaffected at 3, 8 and 12 months post-partum, as tested using Morris water maze and NOR (Trigiani et al., 2021). Given that the R^+^A^+^ mouse model of PE did not demonstrate impaired hippocampal function several months post-partum suggests that the differing mechanisms used to induce the PE models likely contributes to the discrepancy in findings. Thus, it is possible that the renin-angiotensin system is not involved in the cognitive decline occurring after PE, but rather highlights a role for maternal dyslipidemia.

Diet is an important risk factor for cognitive decline, and those rich with fats and cholesterol are known to impair hippocampal-dependent memory, hippocampal neurogenesis and cause blood-brain barrier dysfunction (Freeman and Granholm, 2012; Abo El- Khair et al., 2014; Ledreux et al., 2016; López-Taboada et al., 2020). In the current study, post-partum ePE rats remained on the 2% cholesterol diet for the duration of the study (∼ 6 months). A limitation is that we did not compare post-partum ePE rats that remained on the diet to those switched back to regular chow. This limitation was due to unexpected neurological complications, specifically spontaneous seizures that occur when post-partum rats are switched from high cholesterol chow back to regular rat chow. However, it allowed us to model the cardiovascular disease and hypertension that is commonly present in women after PE. Here, we show that 2% cholesterol diet during and after pregnancy causes HA dysfunction that may represent an underlying mechanism of PE-induced memory dysfunction that occurs later in life. This study also provides novel information and broadens our understanding of how diets rich in cholesterol impact the hippocampus and its vasculature that is relevant to other diet-related pathologies known to increase dementia risk, including diabetes and obesity.

Chronic hypertension is known to accelerate age-related cognitive decline and directly affect the hippocampus and its vasculature (Yano et al., 2017; Walker et al., 2019; Johnson et al., 2020). In the current study, we expand our understanding of this model of ePE that not only has elevated blood pressure during pregnancy (Schreurs et al., 2013), one of the hallmark symptoms of PE, but also that these rats that had ePE continue to be hypertensive well into the post-partum period. While the direct role of ePE-induced hypertension later in life was not determined in the current study, it was associated with hippocampal vascular dysfunction and impaired memory. We recently showed that ePE-induced HA dysfunction during pregnancy was due to oxidative stress, and that treatment with the anti-oxidant apocynin during ePE restored HA endothelial function (Cipolla et al., 2022). However, determining individual or combined roles for oxidative stress, hypertension and lipids as underlying mechanisms of the prolonged HA dysfunction and memory decline after ePE requires further investigation.

## Conclusion

The arterioles supplying the hippocampus are particularly important in maintaining neurocognitive health given this vulnerable brain region’s central role in learning, memory and cognition and its high susceptibility to hypoxic/ischemic damage. Understanding the function and structure of HAs is important as these vessels may have a role in pathological conditions associated with memory loss and dementia, including PE. Here, we show that this model of ePE mimics some of the cognitive decline that occurs after PE and represents a useful tool to study mechanisms by which PE may cause memory dysfunction and early-onset dementia. Overall, the current study provides new insight and advances our understanding of long-lasting and potentially progressive effects of PE on the hippocampus and its vasculature that may represent a novel vascular mechanism of cognitive decline associated with PE.

## Data Availability

The raw data supporting the conclusions of this article will be made available by the authors, without undue reservation.
